# A Multi-Breed GWAS for Carcass Weight in Jeju Black Cattle and Hanwoo × Jeju Black Crossbreds

**DOI:** 10.3390/biology14121699

**Published:** 2025-11-28

**Authors:** Miyoung Won, Jongan Lee, Sang-Min Shin, Seung-Eun Lee, Won-Jae Kim, Eun-Tae Kim, Tae-Hee Kim, Hee-Bok Park, Borhan Shokrollahi

**Affiliations:** 1 Subtropical Livestock Research Center, National Institute of Animal Science, RDA, Jeju 63242, Republic of Korea; mywon87@korea.kr (M.W.); adamrib@korea.kr (S.-M.S.); selee81@korea.kr (S.-E.L.); wonj1140@korea.kr (W.-J.K.); etkim77@korea.kr (E.-T.K.); 2 Animal Breeding and Genetics Division, National Institute of Animal Science, RDA, Cheonan 31041, Republic of Korea; amasss@korea.kr; 3 Department of Animal Resources Science, Kongju National University, Yesan 3249, Republic of Korea; akflssi@smail.kongju.ac.kr (T.-H.K.);

**Keywords:** carcass weight, Jeju Black cattle, Hanwoo × Jeju Black crossbreds, genome-wide association study, candidate genes, KEGG pathways

## Abstract

Carcass weight (CW) is a major determinant of beef yield and market value in Korea, yet the genetic basis of this trait remains largely unexplored in cattle from Jeju Island. In this study, we performed a genome-wide association study (GWAS) using both a mixed linear model (MLM) and the FarmCPU approach, followed by pathway and network analyses to identify loci and biological functions underlying CW variation. A total of 256 Jeju cattle (92 Jeju Black and 164 Jeju Black × Hanwoo crossbreds) were initially sampled. One crossbred sample failed genotyping, leaving 255 animals (92 Jeju Black and 163 crossbreds) for analysis. Animals were genotyped using the Illumina BovineSNP50 v3 BeadChip, and 39,055 high-quality single nucleotide polymorphisms (SNPs) were retained after quality control. The MLM analysis detected no genome-wide significant associations, whereas the FarmCPU analysis identified six significant loci on *Bos taurus* chromosomes 3, 5, 6, 10, and 13, each explaining 2.55–9.58% of the phenotypic variance. Candidate genes located near these loci included *EIF2B3*, *HECTD3*, *SOX5*, *KLF6*, *PHACTR3*, and two uncharacterized protein-coding genes. Functional enrichment analysis identified biologically relevant pathways including lysine degradation, tryptophan metabolism, glycerolipid metabolism, fatty acid biosynthesis, extracellular matrix–receptor interaction, and signaling cascades such as PI3K–Akt and Rap1, although most pathways were not statistically significant after FDR correction. Protein–protein interaction (PPI) network analysis using STRING highlighted modules of signaling, extracellular matrix, and metabolic genes. These clusters suggest that coordinated interactions among these pathways contribute to carcass growth and development. These findings provide new insights into the molecular basis of CW in Jeju Black and Hanwoo × Jeju Black crossbred cattle and identify candidate genes and pathways that may be useful for genomic selection and the sustainable improvement of Jeju Black cattle populations.

## Simple Summary

Carcass weight is one of the most important traits determining beef yield and economic value in Korea. Jeju Black cattle are a native breed valued for their unique meat quality, but their smaller body size limits productivity. To better understand the genetic factors that influence carcass weight, we analyzed DNA from Jeju Black cattle and Jeju Black × Hanwoo crossbreds using a genome-wide association study (GWAS). We identified several genomic regions and genes that may affect carcass growth, including genes related to skeletal development, muscle formation, and metabolism. These findings provide new genetic information that can support breeding programs aimed at improving carcass yield while preserving the unique characteristics of Jeju Black cattle. The results also help establish a scientific foundation for the sustainable conservation and utilization of this important Korean native breed.

## 1. Introduction

Carcass traits are major determinants of productivity and profitability in beef cattle. Among them, carcass weight (CW) is particularly important because it directly influences yield grade, consumer preference, and market price. In Korea, the national beef grading system evaluates carcass yield primarily on CW, ribeye area, and backfat thickness, with CW being the most influential economic trait [1,2,3]. Consequently, improving CW through genetic selection is a key objective in the Korean beef sector [4,5,6].

Jeju Black cattle, locally termed Heukwoo, are indigenous to Jeju Island and represent a valuable but underutilized genetic resource. Historically recognized for their unique meat quality and cultural significance, they are currently conserved as a Korean Natural Monument [7,8]. However, their smaller body size and slower growth rates relative to Hanwoo, the dominant Korean beef breed, have limited their commercial competitiveness [9]. Recent studies indicate that Jeju Black beef contains higher levels of umami-related amino acids and favorable flavor compounds compared with Hanwoo [10,11]. To integrate these desirable sensory traits with the superior growth performance of Hanwoo, Jeju Black × Hanwoo crossbreds (locally termed Heukhanu), have been developed and now serve both as a practical production population and a valuable genetic resource.

Genetic improvement of carcass traits requires identification of the underlying genomic regions and causal variants. Previous studies in Hanwoo and other beef cattle populations have reported moderate to high heritability for CW (0.30–0.60) and identified numerous quantitative trait loci (QTL) affecting growth and carcass composition [12,13]. Well-established regions include NCAPG–LCORL region on BTA6, associated with growth and skeletal development [14,15]; *CAST* (calpastatin) and *DGAT1* (diacylglycerol O-acyltransferase 1), which influence tenderness and fat deposition; and *GHRH* (growth hormone-releasing hormone), a regulator of growth hormone activity [16,17]. These results highlight the polygenic nature of carcass traits and the importance of continuing genomic studies in diverse cattle populations.

Previous GWASs in beef cattle have repeatedly identified major loci influencing growth and carcass traits, including the NCAPG–LCORL region on BTA6 [14,15], PLAG1 on BTA14 [12,13], and additional genes such as *CAST*, DGAT1, and *GHRH*, which contribute to variation in tenderness, fat deposition, and endocrine regulation [16,17]. These well-characterized loci provide a useful comparative framework for interpreting associations detected in Jeju Black-based populations.

Genome-wide association studies (GWASs) using high-density single nucleotide polymorphism (SNP) arrays are widely used to dissect complex traits in livestock [18]. Multi-locus methods like the FarmCPU (Fixed and Random Model Circulation Probability Unification) have improved statistical power and reduced false positives compared with traditional mixed linear models [19,20,21]. Subsequent functional annotation of GWAS signals via pathway enrichment and protein–protein interaction (PPI) analysis enables biological interpretation of detected loci and for carcass traits, these analysis have emphasized roles for amino acid metabolism, lipid biosynthesis, extracellular matrix remodeling, PI3K–Akt and Rap1 signaling pathways [22,23,24].

Although Jeju Black cattle have been characterized for genetic diversity and genomic estimated breeding values [25,26], no GWAS has examined CW in Jeju Black-based populations. Because Jeju Black and Jeju Black × Hanwoo crossbreds show limited genetic differentiation, they can be studied as a single Jeju Black-based population. Given the economic importance of CW and the conservation and production value of Jeju Black, elucidating the genetic basis of CW in this population is both scientifically relevant and practically significant. Therefore, the objective of this study was to identify genomic regions, positional candidate genes, and biological pathways associated with CW in Jeju Black-based populations using GWAS.

## 2. Materials and Methods

### 2.1. Animal Population and Phenotypic Data

We initially enrolled 256 Jeju Black-based cattle, comprising 92 pure Jeju Black and 164 Jeju Black × Hanwoo crossbred animals. One crossbred sample failed genotyping quality control, resulting in 255 animals (92 Jeju Black, 163 crossbreds) included in the final analysis.

The animals were fattened on different farms across Jeju Island, reflecting the typical production environment for Jeju Black and crossbred cattle. Management conditions, including feed type, feeding duration, and housing, varied somewhat between farms; however, all animals were raised under the standard Hanwoo/Jeju Black fattening system, in which cattle are fed a concentrate-based diet supplemented with roughage and finished for approximately 26–32 months before slaughter. Information on farm location and management batch was recorded and used to verify that animals were fattened under comparable production standards.

All animals were slaughtered between April 2022 and May 2023 at the Jeju Livestock Cooperative slaughterhouse in the Jeju Special Self-Governing Province, following uniform grading by the Korean Institute for Animal Products Quality Evaluation (KAPE). Longissimus dorsi muscle tissue samples were collected during carcass grading and used for genomic DNA extraction.

The study population consisted of 127 steers (castrated males; mean slaughter age 37 months) and 128 cows (females; mean slaughter age 63 months). The analyzed phenotype was carcass weight (CW, kg), recorded as hot carcass weight at slaughter according to the Korean beef grading system. The normality of CW distribution was assessed using the Ryan–Joiner test implemented in Minitab 14 (Minitab Inc., State College, PA, USA), followed by basic descriptive statistical analyses.

### 2.2. DNA Extraction and Genotyping

Genomic DNA was extracted from muscle tissue samples collected at slaughter using a modified salting-out method [27]. DNA concentration and purity were measured with a NanoDrop 2000 spectrophotometer (Thermo Scientific, Waltham, MA, USA). Working aliquots were prepared in TE buffer (10 mM Tris-HCl, pH 7.4; 1 mM EDTA) and stored at −20 °C until further analysis. DNA integrity was additionally confirmed by agarose gel electrophoresis.

In total, 256 animals were initially sampled for genotyping. One crossbred animal failed genotyping due to low call rate, resulting in 255 samples that passed DNA quality thresholds and were successfully genotyped using the Illumina BovineSNP50 v3 BeadChip (Illumina Inc., San Diego, CA, USA), originally based on the ARS-UCD1.3 assembly. The complete sample flow, including animal- and SNP-level QC, is illustrated in Supplementary Figure S1. After quality control, SNP positions were updated and remapped to the *Bos taurus* ARS-UCD1.3 reference genome for annotation and pathway analysis, ensuring consistency with the latest Ensembl resources. All 255 samples passed DNA quality thresholds and were successfully genotyped.

### 2.3. Quality Control of Genotype Data

Genotype data were processed using PLINK v1.9. [28]. SNPs with a minor allele frequency (MAF) < 0.05, Hardy–Weinberg equilibrium (HWE) *p*-value < 1 × 10^−6^, or call rate < 90% (genotyping error > 10%) were removed. Individuals with >10% missing genotypes were also excluded; all 255 genotyped animals passed individual-level QC. After filtering, a total of 39,055 high-quality SNPs were retained. SNP positions were mapped to the *Bos taurus* ARS-UCD1.3 reference genome [29].

### 2.4. Population Structure and Relatedness

To investigate potential population stratification arising from merging 92 Jeju Black and 163 crossbreds into a single population, we performed principal component analysis (PCA). Principal components (PCs) were calculated using PLINK v1.9 [28]. The top three principal components (PC1–PC3) were visualized in R (tidyverse 2.0.0 and rgl packages 1.3.31) and are shown in Supplementary Figure S2. The PCA indicated that Jeju Black and crossbred cattle clustered together without clear separation, suggesting a largely shared genetic background. This observation is consistent with previous genomic studies reporting low genetic differentiation between Jeju Black and Hanwoo-derived populations (FST typically < 0.05), which supports their close genetic relationship and further justifies combining the two groups for joint GWAS analysis. For this reason, both groups were analyzed jointly as a Jeju Black-based population. To account for any subtle structure, PC1–PC3 were included as covariates in the GWAS models, in addition to sex, slaughter age, and cattle type. Additionally, the FarmCPU model incorporates a pseudo-QTN-derived kinship matrix in its random-effect component to correct for relatedness and subtle population structure.

### 2.5. GWAS Analysis

GWAS was conducted using the FarmCPU algorithm implemented in the rMVP package in R (1.4.5) [19,29], which consists of a fixed effect model (FEM) and a random effect model (REM). FEM iteratively fits a fixed-effect model to test individual SNPs, with fixed effects including sex (steer or cow), slaughter age (months), type [Jeju Black (Heukwoo) or Jeju Black × Hanwoo crossbred (Heukhanu)] and pseudo-QTNs as covariates. All animals originated from the same research farm (Nanjicheon) and were managed under a unified feeding and housing system; therefore, no additional environmental or farm-level fixed effects (e.g., farm, feeding system, management group) were required in the model.

Pseudo-QTNs, which represent putative quantitative trait nucleotides, were initially set to empty. The REM defines kinship using pseudo-QTNs and iteratively selects the optimal set of pseudo-QTNs while avoiding model overfitting [20]. In addition to FarmCPU, a mixed linear model (MLM) was also applied as a conventional baseline approach, whereas FarmCPU was included to improve statistical power and reduce confounding given the modest sample size.

Multiple testing correction was applied using the Bonferroni method, with genome-wide significance thresholds set at *p* < 0.05/number of SNPs and suggestive thresholds at *p* < 1/number of SNPs [30]. To evaluate test statistic inflation, the genomic inflation factor (λGC) was calculated for both MLM and FarmCPU models.

CW phenotypes were analyzed on the raw scale without log or Box–Cox transformation, as their distribution did not show severe deviations from normality. Fixed effects (sex, slaughter age, and cattle type) and covariates (PC1–PC3) were included directly in the GWAS models; therefore, CW was not pre-adjusted or residualized prior to association testing. Bonferroni correction was used instead of FDR to maintain a conservative genome-wide threshold and minimize false-positive associations, particularly given the modest sample size and the well-established use of Bonferroni in livestock GWASs using ~30–50k SNP panels.

The proportion of phenotypic variance explained by each SNP was calculated as

$${\%Var}_{SNP}=\frac{2p(1-p)a^{2}}{\delta_{p}^{2}}\times 100$$

where *p* is the minor allele frequency, *a* is the additive effect, and $\delta_{p}^{2}$ is the phenotypic variance of CW, estimated from the null MLM, using the EMMA algorithm [19,29].

To quantify the statistical power of our sample size (*n* = 255), we performed a post hoc power calculation under the additive SNP model. Using the Bonferroni-corrected genome-wide α (1.28 × 10^−6^), phenotype variance of 5095 kg^2^ (SD = 71.38 kg, Table 1), and standard non-central χ^2^ approximation, the minimum detectable variance explained (R^2^) at 80% power was ≈11.2%. This corresponds to additive effects of ~34 kg (MAF = 0.5), ~42 kg (MAF = 0.2), and >~77 kg (MAF = 0.05). Therefore, the present study is well-powered to detect only large-effect loci, while smaller-effect variants may go undetected due to limited sample size.

### 2.6. Identification of Candidate Genes

For each SNP that surpassed the genome-wide or suggestive significance threshold, positional candidate genes were identified using the Ensembl genome browser (https://useast.ensembl.org/Bos_taurus/Info/Index (accessed on 15 November 2024)) with the bovine reference genome assembly ARS-UCD1.3. Genes located within ±100 kb of significant SNPs were considered as positional candidates, with the nearest annotated gene prioritized if no gene was found within this interval. Functional annotations, including gene names and biological roles, were retrieved from the Ensembl database. Only these positional candidate genes were subsequently used as the input list for KEGG pathway and STRING network enrichment analyses.

### 2.7. Functional Enrichment and Pathway Analysis

Candidate genes were defined as all annotated genes located within ±100 kb of the six genome-wide significant SNPs. This set of proximal genes was used as the sole input for downstream enrichment and network analyses. Gene annotation was performed using the *biomaRt* package in R (version 2.48.3) [31,32]. Functional enrichment analysis was performed with the Enrichr database, focusing on the Kyoto Encyclopedia of Genes and Genomes (KEGG) pathway library [33]. To further investigate molecular mechanisms, protein–protein interaction (PPI) networks were constructed using STRING version 12.0 [34]. Unless otherwise noted in the figure legends, STRING analyses were restricted to the input set of positional candidate genes, with network expansion limited to first-shell interactors. Pathways were considered significantly enriched at a Benjamini–Hochberg adjusted *p* < 0.05.

## 3. Results

### 3.1. Phenotypic Measurements

Descriptive statistics of CW for the Jeju Black-based cattle are summarized in Table 1. The study population comprised 127 steers and 128 cows, with mean slaughter ages of 37.1 and 62.7 months, respectively. Mean CW was 405.3 ± 55.89 kg in steers and 326.1 ± 62.85 kg in cows. Across all animals (*n* = 255), CW averaged 365.5 ± 71.38 kg, with a range of 139–526 kg. Both sex and slaughter age were included as fixed effects in subsequent GWAS analyses. The relatively large standard deviation of carcass weight (56–71 kg) reflects biological heterogeneity in the dataset, including variation in sex, slaughter age, and breed composition. These values are comparable to those previously reported for Jeju native cattle and crossbreds [35].

**Table 1:** Descriptive statistics of carcass weight in Jeju Black-based cattle.

Group	N	Age (Months, Mean ± SD)	Carcass Weight (kg, Mean ± SD)	Min (kg)	Max (kg)
Steers	127	37.1 ± 4.68	405.3 ± 55.89	182	526
Cows	128	62.7 ± 37.24	326.1 ± 62.85	139	474
Total	255	50.0 ± 29.46	365.5 ± 71.38	139	526

### 3.2. GWAS and Candidate Gene Identification

Genome-wide association analysis for CW in Jeju Black-based cattle was performed using both MLM and FarmCPU approaches. Prior to GWAS, a total of 53,866 SNPs were available from the GenomeStudio FinalReport, of which 39,055 high-quality SNPs remained after quality control filtering (Table 2).

**Figure 1:**
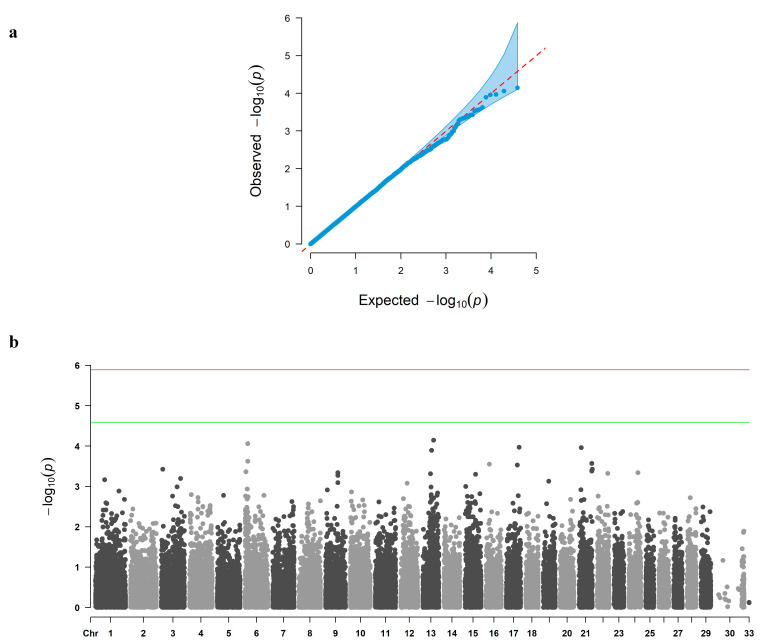
Quantile–quantile (**a**) and Manhattan (**b**) plots of the MLM (mixed linear model) analysis for carcass weight in Jeju Black-based cattle. Chromosomes are BTA 1–29 and X (ARS-UCD1.3); variants mapped to unplaced scaffolds are grouped under “33 (Un)” for plotting convenience. The red line indicates the Bonferroni genome-wide threshold (−log10(0.05/39,055) = 5.89), and the green line indicates the Bonferroni suggestive threshold (−log10(1/39,055) = 4.59). SNPs above the red line are considered genome-wide significant, while SNPs between the red and green lines are suggestively significant. The genomic inflation factor for MLM was λGC = 1.02.

**Figure 2:**
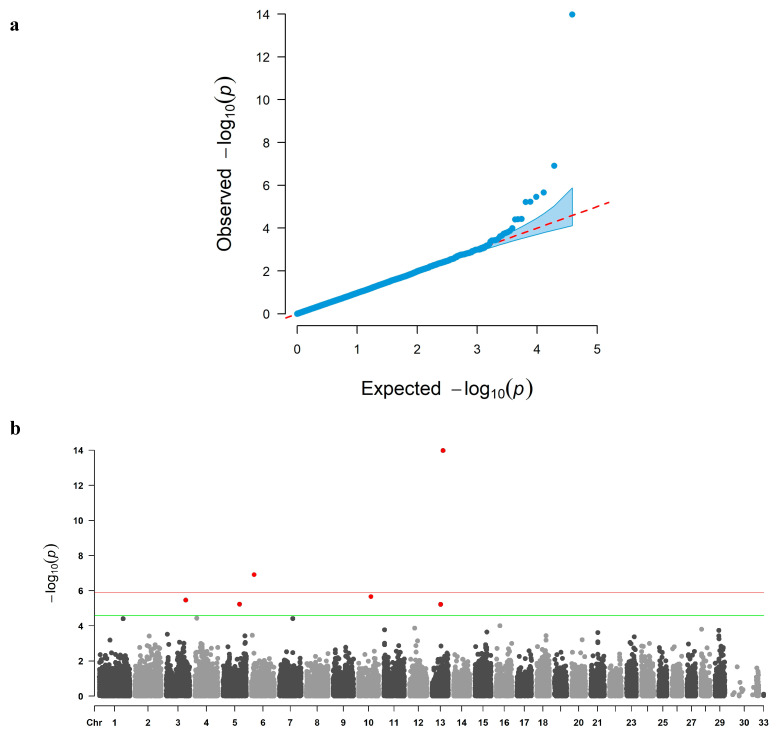
Quantile–quantile (**a**) and Manhattan (**b**) plots of the FarmCPU (fixed and random model circulation probability unification) analysis for carcass weight in Jeju Black-based cattle. Chromosomes are BTA 1–29 and X (ARS-UCD1.3); variants on unplaced scaffolds are shown as “33 (Un)”. The red and green lines indicate the Bonferroni genome-wide (5.89) and suggestive (4.59) thresholds, respectively. SNPs above the red line are considered genome-wide significant, while SNPs between the red and green lines are suggestively significant. The genomic inflation factor for FarmCPU was λGC = 1.04.

The QQ plot from the MLM analysis indicated that the observed distribution of *p*-values closely followed the null expectation (Figure 1a), and no SNPs surpassed the genome-wide significance threshold (Figure 1b). In contrast, the QQ plot from the FarmCPU analysis showed deviation at the tail of the distribution (Figure 2a), and the Manhattan plot revealed six loci surpassing the genome-wide significance threshold on chromosomes 3, 5, 6, 10, and 13 (Figure 2b).

To evaluate potential test statistic inflation, the genomic inflation factor (λGC) was calculated. λGC values were 1.02 for MLM and 1.04 for FarmCPU, indicating minimal inflation and confirming that population stratification was adequately controlled by including PC1–PC3 covariates in the models.

For multiple testing, Bonferroni-corrected thresholds were calculated as −log_10_(0.05/39,055) ≈ 5.89 for genome-wide significance and −log_10_(1/39,055) ≈ 4.59 for suggestive significance. These cutoffs are shown as horizontal lines in the Manhattan plots (Figure 1b and Figure 2b).

The significant SNPs explained between 2.55% and 9.58% of the phenotypic variance in CW (Table 3). Genes located within ±100 kb of these SNPs were identified as positional candidates, including *EIF2B3*, *HECTD3*, *SOX5*, *ENSBTAG00000064813*, *ENSBTAG00000064392*, *KLF6*, and *PHACTR3*. The lead SNPs included markers on *BTA3* (e.g., *SNP_ID1*), BTA5 (*SNP_ID2*), BTA6 (*SNP_ID3*), BTA10 (*SNP_ID4*), and *BTA13* (*SNP_ID5*), which represented the most significant associations detected by the FarmCPU model.

### 3.3. Functional Enrichment and Network Analysis

KEGG pathway enrichment was performed using the positional candidate genes, defined as those located within ±100 kb of genome-wide significant SNPs. This analysis highlighted several nominally enriched biological processes associated with CW. The most biologically relevant but not statistically significant after FDR correction pathways included lysine degradation (9 input genes, *p* = 2.05 × 10^−4^), axon guidance (16 input genes, *p* = 3.46 × 10^−4^), and tryptophan metabolism (6 input genes, *p* = 0.0024). Additional pathways included glycerolipid metabolism, fatty acid biosynthesis, ECM–receptor interaction, mucin-type O-glycan biosynthesis, endocytosis, arrhythmogenic right ventricular cardiomyopathy, ubiquitin-mediated proteolysis, Rap1 signaling, and PI3K–Akt signaling (Table S1). It should be noted that enrichment was calculated solely from the ±100 kb candidate gene set. KEGG outputs, however, list all annotated pathway members overlapping with the input.

**Figure 3:**
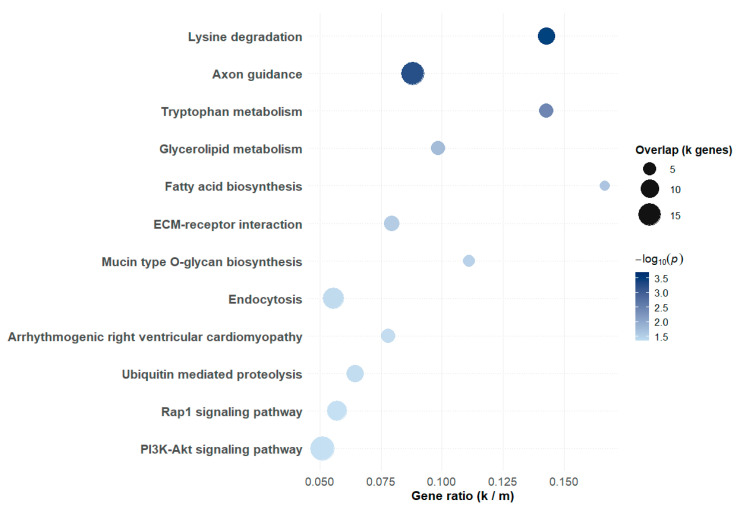
KEGG pathway enrichment bubble plot of positional candidate genes associated with carcass weight in Jeju Black-based cattle. Pathways were ranked by raw *p*-value, and the top 12 are shown. The *x*-axis indicates the gene ratio (number of candidate genes in the pathway divided by total genes in the pathway). The size of each bubble represents the number of overlapping genes (*k*), and the color intensity corresponds to the statistical significance (−log_10_ *p*).

**Figure 4:**
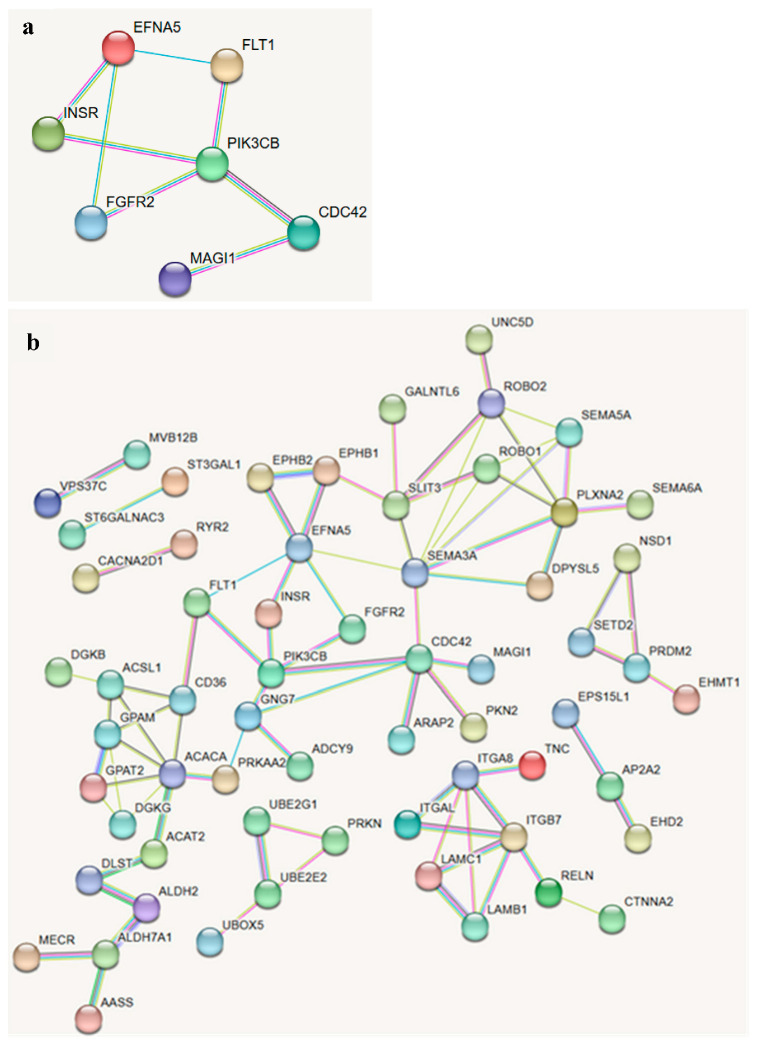
STRING protein–protein interaction (PPI) networks of candidate genes associated with carcass weight in Jeju Black-based cattle. (**a**) High-confidence PPI network (interaction score ≥0.7) of genes from the Rap1 signaling pathway, showing a compact cluster of seven nodes (*PIK3CB*, *EFNA5*, *FLT1*, *INSR*, *FGFR2*, *CDC42*, *MAGI1*). *PIK3CB* acted as the central hub linking receptor tyrosine kinases and downstream regulators. (**b**) Broader PPI network (interaction score ≥0.4) including all positional candidate genes from enriched KEGG pathways. The network revealed distinct modules, including a signaling cluster (*FGFR2*, *CDC42*, *PIK3CB*, *EFNA5*, *FLT1*), an extracellular matrix cluster (*RELN*, *LAMC1*, *LAMB1*, *ITGA8*, *ITGB7*, *TNC*), and a metabolic cluster (*ALDH2*, *ACAT2*, *MECR*, *ACSL1*, *DGKG*, *GPAM*). The colors in the network represent different biological modules, with each module assigned a distinct color for visual clarity.

To provide an overview of these results, the top 12 enriched KEGG pathways are also summarized in a bubble plot, where gene ratio is shown on the x-axis, bubble size reflects the number of overlapping genes, and bubble color represents −log_10_(*p*) (Figure 3). This visualization complements Table S1 by highlighting the relative strength and contribution of each pathway. To explore gene–gene interactions within these pathways, STRING network analysis was performed using candidate genes identified from KEGG enrichment. In the Rap1 signaling pathway, a high-confidence network highlighted a compact cluster of interacting genes including *PIK3CB*, *EFNA5*, *FLT1*, *INSR*, *FGFR2*, *CDC42*, and *MAGI1*, with *PIK3CB* acting as a central hub (Figure 4a). When the interaction strength threshold was relaxed, a broader network including additional Rap1-related genes was observed, capturing more extensive interactions but with lower overall confidence. 

A second STRING network constructed from all candidate genes across nominally enriched pathways revealed multiple interconnected modules (Figure 4b). Prominent clusters included: (i) a signaling module containing *FGFR2*, *CDC42*, *PIK3CB*, *EFNA5*, and *FLT1*; (ii) an extracellular matrix module including *RELN*, *LAMC1*, *LAMB1*, *ITGA8*, *ITGB7*, and *TNC*; and (iii) a metabolic module with *ALDH2*, *ACAT2*, *MECR*, *ACSL1*, *DGKG*, and *GPAM*. These clusters corresponded with KEGG pathways such as *PI3K*–*Akt* signaling, Rap1 signaling, and ECM–receptor interaction.

## 4. Discussion

This study provides the first genome-wide association analysis of CW in Jeju Black-based cattle. Using the FarmCPU algorithm, we identified six significant loci across chromosomes *BTA3*, *BTA5*, *BTA6*, *BTA10*, and *BTA13*. These loci explained between 2.55% and 9.58% of the phenotypic variance, consistent with the highly polygenic nature of CW, where multiple small- to moderate-effect loci act in concert rather than a single major gene [12,36]. Similar results have been reported across beef cattle populations, where genome-wide reviews [37] and meta-analyses across multiple breeds [38] have emphasized the dispersed and multi-locus architecture of carcass and growth traits. Large-scale Hanwoo studies further confirm that CW exhibits moderate heritability and is influenced by multiple QTL across the genome [12]. Comparable findings in other beef populations, such as pasture-fed or Bos indicus-influenced breeds, also demonstrate that CW is shaped by multiple genomic regions rather than a dominant locus [39,40]. These results reinforce the polygenic nature of CW and underscore the value of crossbred populations for identifying additional loci that may not be captured in single-breed studies.

Large-scale GWASs in Hanwoo have consistently identified strong signals on BTA6 (NCAPG–LCORL region) and BTA14 (*PLAG1* locus), including studies with over 7000–9000 animals [12,41]. These loci are widely recognized as the most influential QTL for growth and carcass traits in cattle [15,42]. Although our study was limited by a modest sample size (*n* = 255), the FarmCPU approach still detected a significant locus on *BTA6* close to previously described regions, supporting the robustness of our findings [21]. In addition, we identified associations on *BTA13* that align with reports of reproductive trait loci in Hanwoo [43], as well as signals on *BTA5*, which have not been widely reported in Hanwoo but are consistent with observations in other cattle breeds.

Among our positional candidates, *KLF6* (*BTA13*) has direct evidence in cattle, polymorphisms associated with carcass and body measurements and functional roles in bovine myoblasts and preadipocytes. In contrast, *SOX5* on *BTA5* is a highly plausible skeletal-development gene: it belongs to the SRY-box (*SOX*) family and functions as an HMG-box transcription factor critically involved in chondrogenesis, acting cooperatively with *SOX6* and *SOX9* to activate cartilage matrix genes such as *COL2A1* and *ACAN*, essential steps in chondrocyte differentiation [44,45]. These functions provide a plausible biological pathway linking *SOX5* to variation in carcass growth. Structural variation in *SOX5* has been associated with wither height in Ashidan yak; specifically, *SOX5 CNVs* were significantly linked to height at 18 months [46]. In Hanwoo, wither height itself exhibits moderate positive correlations with CW [47], suggesting that *SOX5* may contribute to CW by influencing skeletal frame size and growth potential.

Another significant locus, ARS-BFGL-NGS-23974 on *BTA13*, was located near *KLF6* (Kruppel-like factor 6). *KLF6* is a member of the Sp1/KLF transcription factor family and is involved in regulating cell proliferation, apoptosis, differentiation, and adipogenesis [48]. In cattle, *KLF6* has been identified within carcass-related QTL in Qinchuan [49] and more recently shown to promote bovine preadipocyte proliferation [50] linking this gene to tissue growth and fat deposition. Comparative sequence analyses confirm that *KLF6* is highly conserved across taurine cattle, indicine breeds, and yak, with expression increasing in adipose, muscle, and visceral organs from calf to adult stages [49]. These observations support *KLF6* as a plausible candidate gene influencing carcass growth in beef cattle. Although our GWAS identified *SOX5* and *KLF6* as biologically plausible candidate genes influencing carcass weight, their functional effects were not directly verified in this study. Future validation through gene expression or functional genomic analyses will be essential to confirm their causal roles and refine their utility as selection markers in Jeju Black-based cattle.

Other candidate genes identified in this study included *EIF2B3, HECTD3,* and *PHACTR3*. Although these genes have not been previously linked to carcass traits, their known roles in translation initiation (*EIF2B3*), ubiquitin-mediated proteolysis, and cytoskeleton regulation suggest potential biological roles in growth (e.g., *EIF2B3* catalyzes GDP-to-GTP exchange on eIF2, a rate-limiting step in translation initiation) [51]. Moreover, two significant SNPs mapped to uncharacterized loci (*ENSBTAG00000064392* and *ENSBTAG00000064813*). At present, their functions are not well characterized, but their genomic positions suggest they may represent novel regulatory elements influencing growth or metabolism. These loci highlight the need for further functional annotation of the bovine genome and suggest that undiscovered regulators of carcass traits may exist.

Functional enrichment analysis suggested biologically relevant pathways including PI3K–Akt signaling, Rap1 signaling, extracellular matrix–receptor interaction, and amino acid metabolism, although these pathways did not remain statistically significant after FDR correction. The identified pathway clusters are consistent with the systems-level organization of coexpressed gene modules observed in large-scale transcriptomic analyses across multiple tissues [52]. The *PI3K*–*Akt* pathway is well established as a regulator of muscle hypertrophy, energy balance, and cell survival, and in bovine muscle satellite cells, IGF1 has been shown to activate *PI3K–Akt–mTOR* signaling to promote proliferation and differentiation [53]. Additional studies demonstrate that myostatin knockdown or FHL3 expression enhances bovine myogenic differentiation via PI3K–Akt signaling, underscoring its importance in muscle fiber growth and protein accretion [54,55]. These functions directly link PI3K–Akt activity to carcass yield and intramuscular fat deposition, traits that strongly influence beef quality and economic value [56]. In our dataset, several components of this pathway were highlighted, including *PIK3CB*, *INSR*, *FGFR2*, *CDC42*, and *EFNA5*. Similarly, enrichment of Rap1 signaling, involving *FLT1* and *CDC42*, reflects its role in cytoskeletal organization and cell adhesion, processes critical for muscle development [57]. Beyond its structural role, Rap1 regulates integrin activation and actomyosin assembly, which contribute to muscle fiber integrity, postmortem tissue remodeling, and ultimately meat tenderness [58,59].

Extracellular matrix remodeling also emerged as an important process, with genes such as *RELN*, *LAMC1*, *LAMB1*, *ITGA8*, *ITGB7*, and *TNC* forming strong interaction clusters. ECM integrity and turnover have long been recognized as major factors influencing muscle structure and meat tenderness [60]. Enrichment of lysine and tryptophan metabolism is consistent with the established requirement of amino acid supply for protein accretion in skeletal muscle [61], and prior cattle metabolomics link lysine (including lysine-degradation intermediates) to feed efficiency and tryptophan to beef quality [62,63,64].

Together, these results highlight both known and novel biological processes underlying CW variation in cattle. The Jeju Black-based population provides a unique genetic background, combining the flavor and cultural value of Jeju Black cattle with the production traits of Hanwoo. The loci and pathways identified here may therefore serve as useful targets for genomic selection, particularly markers near SOX5 and KLF6, which have potential to improve CW while preserving breed-specific characteristics. Recent genomic selection efforts in Hanwoo using single-step marker effect and ssGBLUP models have demonstrated improved accuracy over conventional approaches for carcass traits, including carcass weight, eye muscle area, backfat, and marbling [65]. Similarly, the use of ssGBLUP in Hanwoo increased prediction accuracy for primal cut yields relative to pedigree-based BLUP [66].

Although the present study focused solely on carcass weight, this approach is consistent with the established progression of genomic research in beef cattle, where single-trait GWASs serve as foundational analyses to identify large-effect loci prior to integration into multi-trait genomic selection frameworks. Previous single-trait GWASs in Japanese Black and Hanwoo populations have successfully detected major QTL on BTA14 near the *PLAG1–CHCHD7* region and on BTA6 encompassing *NCAPG–LCORL*, which remain among the most influential loci for carcass weight and growth traits [21,67,68,69]. More recently, Adhikari, Kantar [21] confirmed these genomic regions and discovered additional CW-associated genes (*EIF5*, *LYPLA1*, *MRPL15*) in pasture-finished cattle, demonstrating the robustness of single-trait analyses across diverse production systems. Collectively, these studies reinforce that focused single-trait GWASs remain crucial for identifying major candidate genes that form the basis for multi-trait genomic prediction and pleiotropy-aware selection models. Accordingly, the loci reported here provide a valuable genetic foundation for extending genomic selection in Jeju Black and Hanwoo crossbred populations to multi-trait frameworks encompassing other carcass and quality attributes.

Although this GWAS identified biologically plausible candidate genes related to skeletal growth, adipogenesis, and metabolic regulation, experimental validation of their effects was beyond the scope of this study. Future expression, transcriptomic, or genome-editing analyses are needed to confirm causality and assess potential pleiotropic influences on other carcass and meat-quality traits, thereby refining their value for genomic selection in beef cattle.

Nevertheless, this study had limitations. The modest sample size (*n* = 255) and the use of a 50K SNP array reduce statistical power and resolution, restricting the discovery of small-effect loci. This limitation primarily reflects the inherently small population size of Jeju Black cattle, a rare native breed raised exclusively on Jeju Island and maintained under limited conservation herd [25]. The animals analyzed here therefore represent nearly the entire available genotyped population, providing a valuable yet finite genomic resource for this breed. Although a limited cohort may increase the likelihood of inflated effect size estimates, the use of the FarmCPU algorithm effectively improved detection power and controlled false positives, while the low genomic inflation factors (λGC = 1.02 for MLM and 1.04 for FarmCPU) indicate minimal stratification bias. Despite the small population size, several significant loci detected in this study overlap with genomic regions previously reported in larger Hanwoo and Japanese Black populations [67,68,69], reinforcing the biological credibility of our findings. Although FarmCPU improved power compared with standard MLM, replication in larger populations and use of higher-density SNP panels or whole-genome sequencing are necessary to refine QTL intervals and identify causal variants [19,70]. Functional validation of genes such as *SOX5* and *KLF6* through gene expression, transcriptomics, or genome editing would strengthen causal inference. Another limitation is that we focused only on carcass weight, whereas additional carcass and meat quality phenotypes (e.g., ribeye area, marbling score, backfat thickness) would provide a more comprehensive view of the genetic architecture of beef production. Future research should extend beyond CW to include traits such as ribeye area, marbling score, and backfat thickness, which substantially influence beef market value by affecting yield grade, meat quality, and consumer preferences [39,71].

Moreover, future validation of the candidate genes, including SOX5, KLF6, EIF2B3, and PHACTR3, in larger populations and across breeds using targeted genotyping assays (e.g., KASP or custom SNP panels), gene expression analyses (e.g., RT-qPCR or RNA-seq), or functional studies (e.g., genome editing in bovine cells) would be valuable to confirm their causal roles and assess their utility for genomic selection programs.

## 5. Conclusions

This study provides the first genome-wide association analysis of carcass weight in Jeju Black-based cattle. Using the FarmCPU algorithm, we identified six significant loci across five chromosomes explaining 2.55–9.58% of the phenotypic variance. Candidate genes highlighted included SOX5, KLF6, *PHACTR3*, *EIF2B3*, *HECTD3*, and two uncharacterized loci. Functional enrichment analysis revealed pathways related to *PI3K–Akt* signaling, *Rap1* signaling, extracellular matrix–receptor interactions, and amino acid metabolism, emphasizing the complex, polygenic nature of carcass weight regulation. These findings not only expand the current understanding of carcass weight genetics but also hold practical significance for beef cattle breeding in Korea. The identified loci and candidate genes provide valuable genomic resources for marker-assisted and genomic selection strategies aimed at improving carcass yield and growth efficiency. In particular, variants near *SOX5* and *KLF6* could serve as promising targets for enhancing genomic estimated breeding values (GEBVs) within national Hanwoo and Jeju Black cattle improvement programs. Moreover, these results contribute to the establishment of a genomic foundation for sustainable breeding and conservation of the Jeju Black cattle population, a unique indigenous genetic resource in Korea.

## Figures and Tables

**Table 2:** Summary of SNP quality control.

QC Stage	Number of SNPs
Before QC (GenomeStudio FinalReport)	53,866
After QC (final GWAS dataset)	39,055

**Table 3:** Significant SNPs associated with carcass weight in Jeju Black-based cattle identified by FarmCPU genome-wide association analysis.

CHR	SNP ID	POS	REF	ALT	Effect	SE	*p*-Value	%Var	Positional Candidate Gene
13	ARS-BFGL-NGS-21065	56,698,060	G	A	25.0962	3.317	1.06 × 10^−14^	9.58	*PHACTR3*
6	ARS-BFGL-NGS-116085	14,038,382	A	C	10.3934	3.827	1.22 × 10^−07^	4.54	*ENSBTAG00000064813*
10	ARS-BFGL-BAC-14182	64,224,480	A	C	–18.8834	4.246	2.17 × 10^−06^	4.02	*ENSBTAG00000064392*
3	Hapmap51970-BTA-100380	101,076,596	A	G	22.4000	5.918	3.46 × 10^−06^	2.55	*EIF2B3, HECTD3*
5	BTA-74501-no-rs	86,142,655	G	A	–13.0463	3.537	5.81 × 10^−06^	2.86	*SOX5*
13	ARS-BFGL-NGS-23974	44,467,210	C	A	14.4027	3.613	6.05 × 10^−06^	3.30	*KLF6*

CHR = chromosome number; SNP ID = single nucleotide polymorphism identifier; Position (bp) = physical location on the ARS-UCD1.3 bovine reference genome; REF = reference allele; ALT = alternative allele; Additive effect = estimated effect of the ALT allele on carcass weight (kg); SE = standard error of the additive effect; %Var = percentage of phenotypic variance explained by the SNP; candidate genes were assigned based on the nearest annotated gene within ±100 kb of the significant SNP.

## Data Availability

The data supporting the results of this study are available on request from the corresponding author. Genotype data are not publicly available due to privacy restrictions from the Rural Development Administration.

## Supplementary Materials

The following supporting information can be downloaded at https://www.mdpi.com/article/10.3390/biology14121699/s1, Table S1. KEGG pathways nominally enriched among positional candidate genes (±100 kb from significant SNPs) associated with carcass weight in Jeju Black-based cattle. Pathways were identified using the Enrichr database. “Genes” column lists overlapping candidate genes present in each pathway, as annotated in KEGG. *P*-values were adjusted using the Benjamini–Hochberg method. Figure S1. Sample-flow diagram of animals and SNPs used in the study. A total of 256 cattle (92 Jeju Black and 164 Jeju Black × Hanwoo crossbreds) were initially sampled. One crossbred failed genotyping due to a low call rate, leaving 255 animals (92 Jeju Black and 163 crossbreds) that passed individual-level QC. After SNP filtering based on minor allele frequency (MAF < 0.05), Hardy–Weinberg equilibrium (HWE *p* < 1 × 10^−6^), and call rate (<90%), 39,055 high-quality SNPs were retained for GWAS analyses using MLM and FarmCPU models. Figure S2. Three-dimensional PCA plots (PC1–PC3) of Jeju Black (Heukwoo) and Jeju Black × Hanwoo crossbred (Heukhanu) cattle. The two groups show highly overlapping genetic structure without clear separation, supporting their treatment as a single Jeju Black–based population in downstream analyses.
