# Supplementary material for: A Multi-Breed GWAS for Carcass Weight in Jeju Black Cattle and Hanwoo × Jeju Black Crossbreds
Source: Biology (Basel). 2025 Nov 28;14(12):1699. doi: 10.3390/biology14121699 (PMC12729918; doi:10.3390/biology14121699)
Supplement: Supplementary file 1 [file biology-14-01699-s001.zip › biology-3981831-supplementary.pdf]

Table S1. KEGG pathways nominally enriched among positional candidate genes (±100 kb from significant SNPs) associated with carcass weight in Jeju Black-based cattle. Pathways were identified using the Enrichr database. "Genes" column lists overlapping candidate genes present in each pathway, as annotated in KEGG. *P*-values were adjusted using the Benjamini–Hochberg method.

KEGG Pathway	Overlap (k/m)	*P*-value	Adjusted *P*-value	Odds Ratio	Combined Score	Genes
Lysine degradation	9/63	2.05E-04	4.72E-02	4.96	42.14	SETD2; ALDH2; NSD1; EHMT1; DLST; PRDM2; ALDH7A1; AASS; ACAT2
Axon guidance	16/182	3.46E-04	4.72E-02	2.88	22.98	SEMA5A; ROBO2; SEMA6A; SEMA3A; NFATC3; LRRC4; UNC5D; PIK3CB; EFNA5; ROBO1; CDC42; DPYSL5; PLXNA2; SLIT3; EPHB2; EPHB1
Tryptophan metabolism	6/42	2.35E-03	2.14E-01	4.94	29.92	DDC; ALDH2; DLST; IDO2; ALDH7A1; ACAT2
Glycerolipid metabolism	6/61	1.48E-02	9.23E-01	3.23	13.62	DGKG; ALDH2; GPAM; DGKB; GPAT2; ALDH7A1
Fatty acid biosynthesis	3/18	1.99E-02	9.23E-01	5.91	23.14	MECR; ACSL1; ACACA
ECM–receptor interaction	7/88	2.57E-02	9.23E-01	2.56	9.38	RELN; TNC; ITGA8; ITGB7; LAMB1; LAMC1; CD36
Mucin type O-glycan biosynthesis	4/36	2.96E-02	9.23E-01	3.70	13.01	GALNT14; GALNTL6; ST6GALNAC3; ST3GAL1
Endocytosis	14/252	3.94E-02	9.23E-01	1.75	5.65	ARFGEF2; ZFYVE9; IQSEC1; ARAP2; VPS37C; EPS15L1; ASAP2; AP2A2; CDC42; EHD2; GRK5; MVB12B; RAB11FIP4; FGFR2
Arrhythmogenic right ventricular cardiomyopathy	6/77	4.09E-02	9.23E-01	2.50	8.00	RYR2; SGCD; CACNA2D1; ITGA8; ITGB7; CTNNA2
Ubiquitin mediated proteolysis	9/140	4.16E-02	9.23E-01	2.04	6.48	PIAS4; PRKN; KLHL9; UBOX5; UBE2E2; UBE2G1; WWP2; RCHY1; BIRC2
Rap1 signaling pathway	12/210	4.51E-02	9.23E-01	1.80	5.57	MAGI1; CDC42; ADCY9; FLT1; INSR; MAGI2; PIK3CB; EFNA5; ITGAL; SIPA1L3; SIPA1; FGFR2
PI3K–Akt signaling pathway	18/354	4.54E-02	9.23E-01	1.59	4.93	MAGI1; CSF3R; FLT1; PRKAA2; INSR; MAGI2; TNC; LAMB1; LAMC1; PIK3CB; EFNA5; RELN; GNG7; ITGA8; PKN2; ITGB7; FGFR2; CREB5

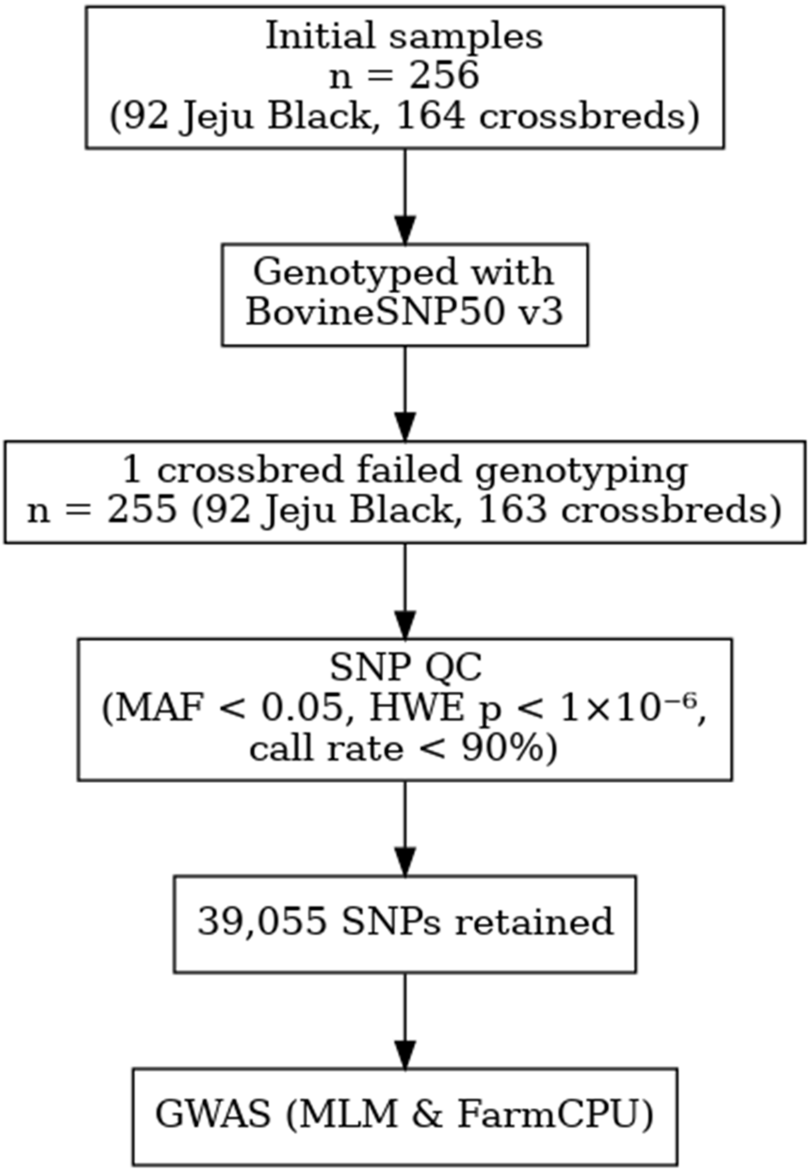

Figure S1. Sample-flow diagram of animals and SNPs used in the study. A total of 256 cattle (92 Jeju Black and 164 Jeju Black × Hanwoo crossbreds) were initially sampled. One crossbred failed genotyping due to a low call rate, leaving 255 animals (92 Jeju Black and 163 crossbreds) that passed individual-level QC. After SNP filtering based on minor allele frequency (MAF < 0.05), Hardy–Weinberg equilibrium (HWE $p < 1 \times 10^{-6}$), and call rate (<90%), 39,055 high-quality SNPs were retained for GWAS analyses using MLM and FarmCPU models.

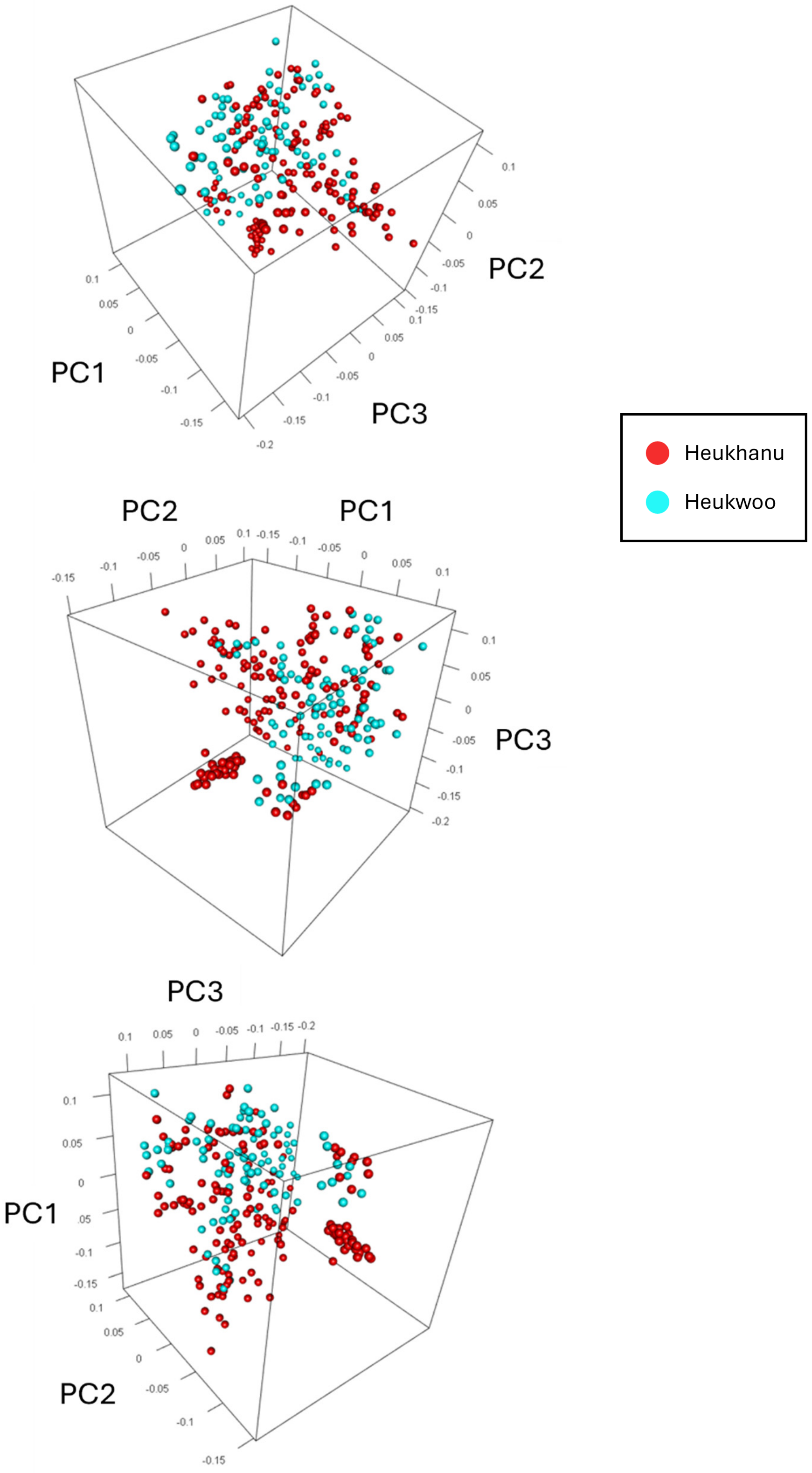

Figure S2. Three-dimensional PCA plots (PC1–PC3) of Jeju Black (Heukwoo) and Jeju Black × Hanwoo crossbred (Heukhanu) cattle. The two groups show highly overlapping genetic structure without clear separation, supporting their treatment as a single Jeju Black–based population in downstream analyses.
